# Chemical Genetics — A Versatile Method to Combine Science and Higher Level Teaching in Molecular Genetics

**DOI:** 10.3390/molecules171011920

**Published:** 2012-10-09

**Authors:** Björn Sandrock

**Affiliations:** Department of Biology-Genetics, Philipps-Universität Marburg, Karl-von-Frisch-Str. 8, 35043 Marburg, Germany; Email: sandrock@staff.uni-marburg.de; Tel.: +49-6421-28-27080; Fax: +49-6421-28-28971

**Keywords:** *U. Maydis*, chemical genetics, problem based learning, Don3as

## Abstract

Phosphorylation is a key event in many cellular processes like cell cycle, transformation of environmental signals to transcriptional activation or polar growth. The chemical genetics approach can be used to analyse the effect of highly specific inhibition *in vivo* and is a promising method to screen for kinase targets. We have used this approach to study the role of the germinal centre kinase Don3 during the cell division in the phytopathogenic fungus *Ustilago maydis*. Due to the easy determination of the *don3* phenotype we have chosen this approach for a genetic course for M.Sc. students and for IMPRS (International Max-Planck research school) students. According to the principle of “problem-based learning” the aim of this two-week course is to transfer knowledge about the broad spectrum of kinases to the students and that the students acquire the ability to design their own analog-sensitive kinase of interest. In addition to these training goals, we benefit from these annual courses the synthesis of basic constructs for genetic modification of several kinases in our model system *U. maydis*.

## 1. Introduction

The Bologna Declaration in 1999 started a process for the harmonisation of the architecture of the higher education system in Europe [1]. The aim of this process was to set up three cycles with the qualifications Bachelor’s degree, Master’s degree and Doctoral degree to ensure compatibility in the standards and the quality of higher education in Europe. Along with 34 other countries Germany has signed this declaration in 1999 [1]. For the higher education system in Germany this declaration resulted in a new organisation of the degree programmes. At the Philipps-University of Marburg the “Biology” Bachelor-cycle was started in 2004 and the “Molecular and Cellular Biology” Master-cycle in 2007. As part of the Master module “Molecular characterisation of genes” the practical course “Chemical genetics” was created in 2009.

The concept of research-oriented teaching was invented by Wilhelm von Humboldt almost 200 years ago [2]. Today it is defined as result of an increase of complexity in didactical concepts [3]. One level of these concepts is the “problem-based learning”, which consists mainly of student-centered learning that should stimulate self-directed learning [4,5]. Under the guidance of a moderator (the course leader) students are encouraged to maintain a higher level of motivation during their studies and can see the relevance of learning for future career [5]. We have chosen this concept for the course “Chemical genetics”.

Here I show the outline of the course and some results obtained with the analog-sensitive kinases constructed in the last years together with a brief overview over the use of chemical genetics for the analysis of septum formation in the phytopathogenic fungus *Ustilago maydis* [6,7].

## 2. “Chemical Genetics” as Tool to Study Secondary Septum Formation in *U. maydis*

The chemical genetics method has been developed in the group of Kevin Shokat for the analysis of protein kinases [8]. It is based on the identification of a so-called “gatekeeper”-amino acid in the ATP binding pocket. The mutation of this bulky amino acid to a smaller one like alanine or glycine opens the gate for a specific characterization of this kinase. The use of modified ATP analogues allows then the selective targeting of the kinase either with radioactive labelled benzyl-ATP to identify kinase targets or with PP1-analogues to study the effect of kinase inhibition. 

**Figure 1:**
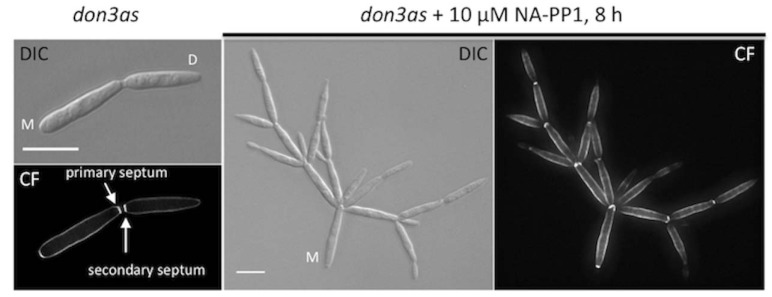
Inhibition of Don3as results in a septation defect. Cells expressing *don3as* were grown to an OD_600_ = 0.1 and split in media with or without inhibitor. After further growth for 8 h (doubling time of *U. maydis*: 2 h) cells were stained with Calcofluor white (CF). The respective brightfield images are shown (DIC, M: mother cell; D: daughter cell). (Scale bars: 10 µm).

This method was used for the characterization of the germinal centre kinase (GCK) Don3 during cytokinesis in the phytopathogenic fungus *Ustilago maydis* [6]. *U. maydis* haploid cells grow by budding at the tip [9,10]. Cell separation occurs by subsequent assembly of a primary and a secondary septum (Figure 1, lower left panel). We were able to demonstrate that formation of secondary septa is cell cycle independent and depends on a Cdc42-module and the Don3 kinase [6]. Don3 was originally identified in a mutagenesis screen for aberrant colony formation [11]. Don3 mutants grow as donut-shaped colonies on low-concentrated agar plates and as huge cell clusters in liquid media. 

The clear phenotype allowed us to further investigate the function of Don3 and therefore we constructed a strain expressing the analog sensitive Don3^M157A^ variant, Don3as. In the presence of the inhibitor NA-PP1 secondary septum formation was immediately blocked, cell separation could not occur and cell clusters were formed (Figure 1) [6]. After the transfer to inhibitor-free medium Don3as became active and secondary septa were assembled simultanously in all cells of the cluster indicating that the function of Don3 is cell cycle independent [6].

With the use of specific proteins involved in septum formation like the actomyosin components Cdc4 and Cdc15, the septin Cdc10 or an actin-binding protein it was possible to get a clear picture of the actomyosin and septin dynamics during septation in *U. maydis* and which of these steps are dependent on Don3 [6,7].

## 3. Chemical Genetics as Appropriate Technique for Higher Education Systems

The M.Sc.-program “Molecular and Cellular Biology” at the Philipps-University Marburg, Germany, was founded in 2007 and has the aim to train students theoretically and methodically in molecular and cell-biologically oriented areas of Biology e.g., in Microbiology, Molecular Genetics, Biochemistry, Cell Biology and Developmental Biology. In our department of Molecular Genetics at the faculty of Biology the students can take two courses, one is located in the first half (seven weeks) of the winter semester and the other in the second half of the summer semester. Each course is separated into a lecture, a seminar and a practical course (for further infomation see [12]). The two-week course “Chemical genetics” is the second of three parts of the practical program of the winter semester on “Molecular Characterization of Genes: From identification to function”, which can be taken by a maximum of 24 students in the first semester. Based on the concept of “problem-based learning” [5] the course combines teaching with actual scientific questions.

The aims of this course are to demonstrate the power of the chemical genetics approach, to attract the students with genetic questions, to strengthen the self-reliance and organization skills of the students and to combine standard molecular and cell biological techniques with project-specific methods. Especially for the latter reason the IMPRS of the Max-Planck-Institute (MPI) in Marburg also accepted this course as a suitable module for its students.

## 4. Structural Organisation of the Course “Chemical Genetics”

The course “Chemical genetics” starts with an introduction unit, in which basic information about protein kinases, the history of chemical genetics and the Ste20-like kinase Don3 from *U. maydis* will be given (Table 1). After that, *U. maydis* protein kinases will be presented, for which the students can generate their own gatekeeper mutation. In a short overview it is important to explain the scientific relevance for analog-sensitive mutants of these selected kinases. Students of the IMPRS can use this module to create their own project specific analog-sensitive kinase using our expertise. Finally, in pairs the students have the task to choose their protein kinase, to identify the gatekeeper amino acid and to design oligonucleotide primers for site directed mutagenesis of the gatekeeper codon together with a nearby silent mutation gaining a restriction site for easier detection of mutants.

**Table 1:** Structural organisation of the course “Chemical genetics”.

Timeline of the course	
Day 1	Full-time seminar Content: What are kinases? What are their functions? What is the concept behind “Chemical genetics”? Problem: Identification of the gatekeeper amino acid in a given kinase or in a kinase of the students choice. Aim: Design of Oligonucleotide-primers for the site-directed mutagenesis of the gatekeeper codon in the kinase of choice and for the left and right borders for homologous recombination in *U. maydis* Formation of 12 groups of two students Presentation of a list of original and review literature Collection and Order of the Primers
Day 2	Time for the students to prepare a colloquium
Day 3	Colloquia of about 30 min for each group Preparation of buffers and media Inoculation of strains
Day 4	PCR for the site-directed mutagenesis of the kinase gene of choice (1) The Don3 kinase in *Ustilago maydis* (2)
Day 5	Cloning of the mutagenized DNA and transformation in *E. coli* (1) Inhibition and release of different analog-sensitive kinase mutants of the last years courses (3)
Day 6	Inoculation of *E. coli* transformants and virtual cloning (1) Microscopy of inhibited and released *don3as* cells surface labelled with Biotin (3)
Day 7	Miniprep, restriction analysis, order for sequences (1)
Day 8	Image processing using e.g., ImageJ open source software (2)
Day 9	Preparation of final talks (1–3), sequence analysis (1)
Day 10	Full-time seminar with each group presenting their results Final discussion

To generate these mutations in *U. maydis* genome by homologous recombination using the SfiI-system four additional primers for the left and right border have to be designed [13]. The designed primers for each protein kinase will be collected, compared, optimized where necessary, and ordered. Due to cost limitations only four kinases per course are chosen. Therefore each kinase is processed by several groups. In most cases this ensured at least one modified gene per kinase and increased the exchange of information, material and products among the students.

After this theoretical and virtual part the practical one follows with three subjects: (1) generation of the mutagenized genes using the two-step PCR approach; (2) repeat of the published *don3as* data and (3) analysis of a mutagenized protein kinases from the years before (Table 1).

We prefer to have the students work in pairs, which increases the social competence as problems and success can be discussed directly. (1) For this project the students have to prepare genomic DNA from *U. maydis*, which is the only species specific step in this part. After that the two-step PCR for the mutagenesis and the PCR for the right border will be performed using individual PCR programs and the corresponding products will be cloned in suitable cloning vectors e.g., the pJET1.2 system from Fermentas [14]. For transformation of ligation products in *E. coli* we propose the use of student-made electrocompetent cells. Overnight cultures of single transformants will be prepared and plasmids of transformants will be isolated. Using suitable restriction enzymes especially the ones which were introduced by the site-directed mutagenesis, the plasmids will be analysed to exclude false positives from sequencing (Supplementary Information, Table S1). Therefore the students should construct their plasmids virtually, which will be a useful personal skill necessary for further genetic studies. (2) To get in contact with the idea of chemical genetics the students reproduce two experiments for the modified Don3 protein kinase, which have been published in Böhmer *et al.* [6]. The first one is to show that the gatekeeper mutation should generate a still active kinase. For Don3 it was evident that the tested mutations M157G and M157A behave differently, and only the latter one is an active variant, which is able to complement the *don3* mutant. The second set of experiments demonstrates that inhibition of Don3as starts directly after the addition of NA-PP1 and cell cluster development can be followed over time. After washing-out of the inhibitor, re-activated Don3as stimulates secondary septum formation, which results in cell cluster dissolution after 30 min. The students also use the biotin cell labeling technique to identify the starting cells during inhibition and release [6]. Each group chooses one time point to analyse the cell cluster stage using fluorescent microscopy. Altogether, in this part of the course the students should learn the basic principles of chemical genetics e.g., mutants should grow like wildtype in the absence of the inhibitor, inhibition should occur directly and the effect of the release should be charaterized.

**Table 2:** Protein kinases used in the “Chemical genetic” courses 2009–2011.

*U. maydis* gene number	Name	Gatekeeper	Observation	Kinase family	Literature
um05543	Don3	M157A	cell separation defect	Germinal centre Kinase	[11]
um10145	Cla4	M629A	polarity defect	p21-activated kinase	[15]
um04956	Ukc1	M398A	morphological defect	NDR-like kinase	[16]
um05698	Nrc2	M454G	no	Phototropin-like kinase	[17]
um10119	Ipl1	M280A	no	PKA-like kinase	[18]
um03841	Ire1	L1082A	no	PKc-like kinase	[19]
um04902	Kin28	L94A	no	Cdk7-like kinase	[20]
um02741	Cbr1	M798A	no	NDR-like kinase	[21]
um11199	Cdc28	M267A	wrong gatekeeper	Cdc2-like kinase	[22]
um11396	Nak1	M769A	no	Germinal centre kinase	[23]
um11396	Nak1	M769G	morphological defect without inhibitor	Germinal centre kinase	[23]

Each year the third part is very exciting for the students and also for the course leader. Their task is to characterize an analog-sensitive variant of a kinase mutagenized in previous years. In the genome of *U. maydis*, 136 genes expressing kinases have been identified (Supplementary Information, Table S2). Many of these have known functions in *U. maydis* or their function can be proposed due to sequence homology to kinases identified in other eucaryotic organisms.

In previous years we have chosen kinases, for which the function could be deduced by sequence similarities to other organisms (Table 2). The gene numbers are from the MUMDB homepage [24]. For Ipl1, Ire1, Kin28, Cdc28 and Nak1 protein names are given from the closest homologs in *S. cerevisiae*. Ukc1 is the closest homolog of yeast Cbk1p and Cbr1 is a Cbk1-related protein. Nrc2 is similar to Nrc-2 kinase from *Neurospora crassa*. The kinase families have been identified using BlastP [25]. 

Using different concentrations, different inhibitors and different media the students have analyzed the effects of kinase inhibition. By this procedure we have been able so far to collect data for gatekeeper mutations of nine kinases (Table 2).

**Figure 2:**
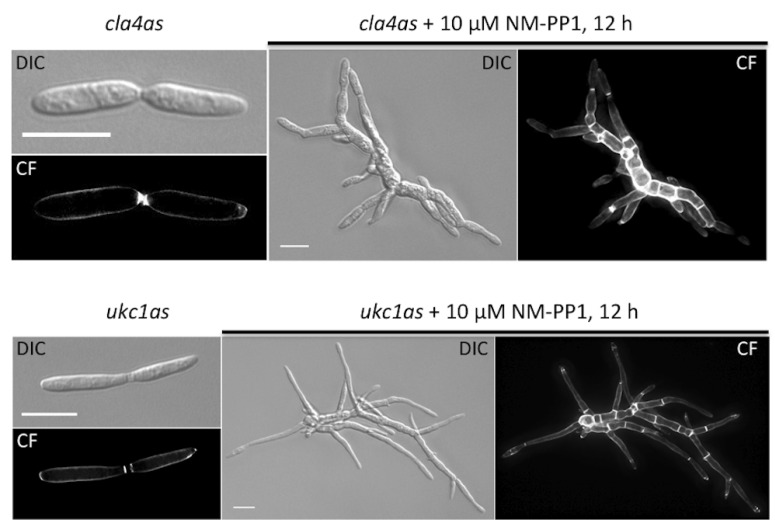
Inhibition of Cla4as and Ukc1as using NM-PP1. Cells expressing *cla4as* of *ukc1as* were grown to an OD_600_ = 0.1 and split in media with or without inhibitor. After further growth for 12 h cells were stained with Calcofluor white (CF). The respective brightfield images are shown (DIC). (Scale bars: 10 µm).

Interestingly, after inhibition with NM-PP1 only two analog-sensitive kinases developed the phenotype of the corresponding deletion mutant: Cla4as, a Ste20-like kinase involved in polar growth [15,26] and Ukc1as, the central NDR kinase regulating morphogenesis also called Cbk1 [16] (Figure 2). No effect could be observed when the kinases Kin28as (CTD kinase of transcription factor TFIIH), Ipl1as (Aurora kinase), Nrc2as (conidiation regulating kinase), Cbr1as (Cbk1-related kinase) and Ire1as (regulator of the unfolded protein response after ER-stress) were inhibited with doses ranging from 10–100 µM of either NA-PP1 or NM-PP1 (see Table 2 for references). 

**Figure 3:**
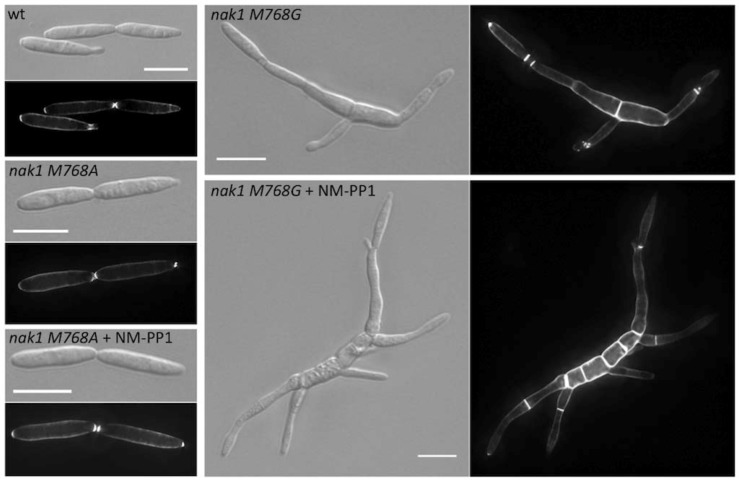
Gatekeeper mutations M768A and M768G in Nak1 did not lead to analog-sensitive kinases. Cells expressing either *nak1 M768A* or *nak1 M768G* were grown to an OD_600_ = 0.1. After addition of the inhibitor cells were grown for 8 h. Representative brightfield images are shown (DIC). (Scale bars: 10 µm).

For Nak1, a germinal centre kinase regulating cell morphogenesis [23], the gatekeeper mutation M768A was also silent, whereas the M768G mutation partially inactivates the kinase activity even without addition of the inhibitor (Figure 3). Cells expressing Nak1^M768G^ showed a defect in cell separation, mislocalized secondary septa and a thickened cell wall. After inhibition with NM-PP1 these effects were more pronounced. Nevertheless this variant was also not suitable.

For the mutated kinase, Cdc28^M267A^, which is a cell cycle regulating kinase of the cyclin dependent kinase family, no effect could be observed. Interestingly, the students in the last year remarked that the mutated amino acid M267A seemed not to be the correct gatekeeper and proposed L264. Therefore, this year students will create a new analog-sensitive variant for L264. The error in the determination of the putative gatekeeper amino acid of Cdc28 derived in that year from too simple sequence alignment analysis using only the kinase domain of Don3 as reference. Like M157 in Don3 the M267 in Cdc28 is followed by glutamate, but the students and I have overlooked that the overall structure of the corresponding subdomains V did not align. L264 in Cdc28 is also followed by glutamate and might be a better candidate being the gatekeeper. To avoid further errors at this stage the students should now use the conserved domain alignment tool of NCBI [25].

As the final practical part the students analyse their sequences and process their microscopic images following guidelines about appropriate and inappropriate manipulations of digital images for scientific publications [27].

Finally, on the last day of the course the students will present their data in a 10 minute talk during a seminar. All results will be collected and discussed especially the observations that some analog-sensitive kinases are not affected by the inhibitor and that some kinases (Nak1 M769G, Don3 M157G) which did not tolerate the gatekeeper mutation might be optimzed by a so-called suppressor of glycin gatekeeper (SOGG)-mutation [28].

## 5. Final Remarks and Future Objectives

For the past three years we have taught this course following the principle of “problem-based learning”. From the teaching side, students get in contact with basic genetics like cloning and mutagenesis, and they learn a method to analyse protein kinases and which problems can occur. In addition, skills are obtained like primer design, microscopic image preparation and presentation of results in a short talk. During the primer design session we always observe that although students know from lectures that DNA is double stranded and each strand has an orientation, many students have problems during the design of the primers with the use of the correct orientation of 5′ and 3′ ends of DNA. If your institute is not equipped with original programs you can use open-source programs like the plasmid editor Ape [29], the image processing and analysis program ImageJ [30] and the presentation program from Apache Open Office [31]. Furthermore, each student deals with at least three different kinases, for which he/she has to read original literature. From the research side we derive from this course mutants which have been or will be analysed further e.g., as a project of a master thesis (Cla4as in [26]). For future courses we want to choose also kinases of unknown function to open up new fields of research.

In feedback given by the students, they mostly stated that they liked the content and the structure of the course. For many of them, it was the first time that they have to coordinate and plan their work mostly by themselves. They also appreciated the use of open-source software, which could be installed also on their home computers. On the other side, students regret that they cannot follow up with their kinase mutants till to the end, but this is due to time limitations during the M.Sc. studies.

## 6. Experimental

### 6.1. Genetic Methods

The generation of the analog-sensitive strains was made according to the protocols described previously [5]. The wild type *U. maydis* strain Bub8 was used [32]. Primers used for the creation of the analog-sensitive kinase genes are listed in Table S1.

### 6.2. Inhibition

For inhibition cells were grown to an OD_600_ = 0,1. The inhibitors 1-(1,1-dimethylethyl)-3-(1-naphthalenyl)-1*H*-pyrazolo[3,4-d]pyrimidin-4-amine (NA-PP1, CAS: 221243-82-9) or 1-(1,1-dimethylethyl)-3-(1-naphthalenylmethyl)-1*H*-pyrazolo[3,4-d]pyrimidin-4-amine (NM-PP1, CAS: 221244-14-0) (Calbiochem, Darmstadt, Germany) dissolved in DMSO were added to a final concentration of 10–50 µM. Cells were grown further for the indicated timepoints. Calcofluor white (CF) (Sigma, Taufkirchen, Germany, #F3543) staining of *U. maydis* cells was performed as described [33].

### 6.3. Microscopy

For differential interference contrast (DIC) and epifluorescence microscopy a Zeiss Axiovert 200 microscope was used (Jena, Germany). Images were taken using a cooled CCD camera (Hamamatsu Orca-ER, Hamamatsu, Japan) with an exposure time of 20–100 ms. Image acquisition was performed using Volocity software (Improvision, PerkinElmer, Waltham, MA, USA). Image analysis and processing was performed using ImageJ (National Institutes of Health, Bethesda, MD, USA).

## 7. Conclusion

The Bologna declaration has rolled up the higher level teaching in Germany. In Molecular Genetics at the Philipps-University of Marburg, Germany, we have started to focus on educational concepts in the restructured Bachelor and Master studies. In this review I have outlined that the concept of “problem-based learning” can be used to combine higher level teaching during the Master studies with actual scientific questions. In the Master course “Chemical genetics” the students learn at multiple levels to create their analog-sensitive kinase using general genetic and molecular biological techniques in the theoretcial background of protein kinase analysis. We are convinced that this way of conceptualisation in higher level teaching will result in a better motivation, a higher qualification and and a greater self-reliance of the students.

## Supplementary Materials

Supplementary materials can be accessed at: http://www.mdpi.com/1420-3049/17/10/11920/s1.
