# Supplementary material for: Chemical Genetics — A Versatile Method to Combine Science and Higher Level Teaching in Molecular Genetics
Source: Molecules. 2012 Oct 9;17(10):11920–30. doi: 10.3390/molecules171011920 (PMC6268829; doi:10.3390/molecules171011920)
Supplement: Supplementary file 1 [file molecules-17-11920-s001.pdf]

Table S1. List of oligonucleotide primers used.

Cla4	LF-5'	GTAGGATCCGCTCTGTCAAGCCTCCGACC
	M629Arev	CCTCCCTCCATGTACTCcgcGATGACCCA*gAGCTC*GTTG
	M629Afwd	CAAC*GAGCTc*TGGGTCATCgcgGAGTACATGGAGGGAGG
	LF-3'	GTAGGCCATCTAGGCCGCAATCTCGTCAAGTAAAGTCG
	RF-5'	GTAGGCCTGAGTGGCCCGAGATTGCAACGTGTAACC
	RF-3'	GTAGGATCCCGTACGCTGCGATCGCTTGC
Ukc1	LF-5'	GCAATATTATGTCTACTTTGAGCG
	M398Arev	CCGCCGGGCAA*gAAtTC*cgcGAGAAGGTACAGATACGc
	M398Afwd	gCGTATCTGTACCTTCTCgcg*GAaTTc*TTGCCCGGCGG
	LF-3'	GAGGCCATCTAGGCCATTTACGATGGCAGACAAAGG
	RF-5'	GTGGCCTGAGTGGCCATTGGTTTGGGCGAATGGC
	RF-3'	GCAATATTCGTACGTCAACAGCGCG
Nrc2	LF-5'	GCAATATTTCGAAAAGGGTCGTTCC
	M454Grev	GCCACCCATGC*AGTAcT*CgccGCAGAGGTAGAGGTAATC
	M454Gfwd	GATTACCTCTACCTCTGCggcG*AgTACT*GCATGGGTGGC
	LF-3'	GAGGCCATCTAGGCCGACGAGTGAAGCTTTCGAGCG
	RF-5'	GAGGCCTGAGTGGCCTAAGCATCTTGGCTTCTGC
	RF-3'	GCAATATTCGGTCAACGCTTTTCAGATACC
Ipl1	LF-5'	GTCAATATTCTACTTTGTGAAGACGCTGC
	M629Arev	GCTCCCCACGACCAGC*gAATTC*GATagcGAGGAAGACTCGGCCCTCATC
	M629Afwd	GATGAGGGCCGAGTCTTCCTCgctATC*GAATTc*GCTGGTCGTGGGGAGC
	LF-3'	TGAGGCCATCTAGGCCGGTGCCTTAGATTCCGTATAGC
	RF-5'	CATGGCCTGAGTGGCCGATTCTTCTTCTGTCATCGAC
	RF-3'	GACAATATTGCTGACCTTGTCTACTTGG
Ire1	LF-5'	GCAATATTAAAGCACAACTCAACGC
	D1014Arev	CCGTAGCCAAGCACCTCGgCC*GAtATc*GTGAGCGAAG
	D1014Afwd	CTTCGCTCAC*gATaTC*GGcCGAGGTGCTTGGCTACGG
	LF-3'	GAGGCCATCTAGGCCAACTGGGCAAAGGAGATGGA
	RF-5'	GAGGCCTGAGTGGCCGTGCGCCTGTGTATCTCTTTG
	RF-3'	GCAATATTGGCCATCTGAGGGCTGAC
Kin28	LF-5'	GACAATATTCATCTTTCACCCTTCCAAAG
	L94Arev	TGATGAGTGCTTCTAGATTGGTGTCggcGAA*cTCgAG*CACCAGGTTG
	L94Afwd	CAACCTGGTG*CTcGAg*TTCgccGACACCAATCTAGAAGCACTCATCA
	LF-3'	TGAGGCCATCTAGGCCCACAGAGATCCGCTTTAATGC
	RF-5'	CATGGCCTGAGTGGCCAGGGCTAGTACGACCTCG
	RF-3'	GACAATATTCGCACCCTGCTAAGATCCC
Cbr1	LF-5'	GTCAATATTGCTCAGGTTCAAGCCGCC
	M798Arev	CTCCTCCTCCCAT*GAAtTC*tgcGATGAGGTAAAGGTGGTCC
	M798Afwd	GGACCACCTTTACCTCATCgca*GAaTTC*ATGGGAGGAGGAG
	LF-3'	TCAGGCCATCTAGGCCAAGTTCAGCTTGACAAGG
	RF-5'	GAGGCCTGAGTGGCCGGGAAGCCTAAAGTCAGG
	RF-3'	GCAATATTCATCTTGGCCGGGTCGTAGG

Table S1. *Cont.*

Cdc28	LF-5'	GTCAATATTTACGAATCGTGAGTTTGTGC
	M267Arev	GCAACGTCTTGAGGTCGTGTTCtgcGAA*cTCgAG*GATAAGGAAGATC
	M267Afwd	GATCTTCCTTATC*CTcGAg*TTCgcaGAACACGACCTCAAGACGTTGC
	LF-3'	TCAGGCCATCTAGGCCTCTGCTATGCCACGTTCTGC
	RF-5'	CATGGCCTGAGTGGCCGACATGTGCGTGGCCGAATC
	RF-3'	GACAATATTCCCAGCACGTCAGCCAAGG
Nak1	LF-5'	GTCAATATTGGTTTCAGAACAGACACC
	M769Arev	GCCACCTTCTGCAAAGTCagcAACAATCCA*gAgCTC*TGGGCCTTTGAGCC
	M769Afwd	GGCTCAAAGGCCCA*GAGcTc*TGGATTGTTgctGACTTTGCAGAAGGTGGC
	LF-3'	GTAGGCCATCTAGGCCCTATCAACCCTAACATCTGGC
	RF-5'	CATGGCCTGAGTGGCCAGGTCCACGGTTGCACACG
	RF-3'	GACAATATTCGCGCTACACCGCGGAAGAGG
Nak1	LF-5'	GCAATATTTGCTGAATCCAACGCTG
	M769Grev	GCCACCTTCTGCAAAGTCtccAACAATCCA*gAgCTC*TGGGCCTTTG
	M769Gfwd	CAAAGGCCCA*GAGcTc*TGGATTGTTggaGACTTTGCAGAAGGTGG
	LF-3'	CAGGCCATCTAGGCCGGAGAAATTAACACAAATAG
	RF-5'	CAGGCCTGAGTGGCCCCTGCCAGATGTTAGGGTTG
	RF-3'	GCAATATTGCCACCCCAAACAGCAG

Lower case nucleotides show the mutations resulting in the analog-sensitive variant. The underlined nucleotides mark the created silent mutations bearing a new restriction site (*italic*).

Table S2. List of *U. maydis* kinases.

No.	Entry	Gene	Description
1	um00109	dak2	probable DAK2 - dihydroxyacetone kinase
2	um00145		related to p53-related protein kinase
3	um00157		probable pyruvate kinase
4	um00215	pro1	probable PRO1 - glutamate 5-kinase
5	um00274		probable casein kinase I
6	um00453		related to phosphatidylinositol 3-kinase
8	um00560		probable glycogen synthase kinase 3 alpha
9	um00584		probable casein kinase-1 hhp1
10	um00602	sch9	probable SCH9 - serine/threonine protein kinase involved in stress response and nutrient-sensing signaling pathway
11	um00691		related to HRK1 - Protein kinase with a role in ion homeostasis
12	um00721	cdc7 (Sp)	related to MAPKK kinase
13	um00760		related to ERG8 - phosphomevalonate kinase
14	um00797		probable adenosine kinase
15	um00957		related to serine/threonine-protein kinase
16	um01033.2		related to VPS15 - ser/thr protein kinase
17	um01110		related to serine-protein kinase atr
18	um01180		probable protein kinase CK2 alpha subunit
19	um01514	fuz7	dual specificity protein kinase Fuz7
20	um01544		related to SSK2 - MAP kinase kinase kinase of the high osmolarity signal transduction pathway

Table S2. *Cont.*

No.	Entry	Gene	Description
21	um01662		related to BCK1 ser/thr protein kinase of the MEKK family
22	um01859		related to ribitol kinase
23	um01962		related to GAL1 - galactokinase
24	um02015		related to uridine kinase
25	um02088	adk1	probable ADK1 - adenylate kinase, cytosolic
26	um02211		related to bifunctional polynucleotide phosphatase/kinase
27	um02244	csr1	related to ser/thr protein kinase Cdc7
28	um02331	kpp6	MAP kinase
29	um02357		probable osmotic sensitive-2 protein (putative mitogen-activated protein (MAP) kinase homolog)
30	um02371		related to serine/threonine protein kinase
31	um02630		hypothetical protein / related to thiamin pyrophosphokinase
32	um02741	cbr1	related to CBK1 - Serine/threonine protein kinase involved in cell wall biosynthesis
33	um02849	guk1	probable GUK1 - guanylate kinase
34	um02967	ynk1	probable YNK1 - nucleoside diphosphate kinase
35	um03081		related to ARK1 - Actin Regulating Kinase
36	um03206		related to thymidylate kinase
37	um03216	tor1	probable TOR1 - 1-phosphatidylinositol 3-kinase
38	um03234.2		related to CDC5 - Serine/threonine-protein kinase
39	um03305	ubc3	MAP kinase
40	um03306	prs4	probable PRS4 - ribose-phosphate pyrophosphokinase 3
41	um03315	ukb1	serine/threonine protein kinase B-related Ukb1
42	um03358		related to SNF1-related protein kinase KIN10
43	um03413		related to Acetate kinase
44	um03446	Sid2	probable protein kinase DBF2
45	um03618		related to THI21 - Hydroxymethylpyrimidine phosphate kinase, involved in the last steps in thiamine biosynthesis
46	um03796		related to dis1-suppressing protein kinase dsk1
47	um03809		related to phosphatidylinositol-4-phosphate 5-kinase
48	um03841	ire1	related to IRE1 - protein kinase
49	um03901		related to Serine/threonine-protein kinase CBK1
50	um03928		related to serine/threonine protein kinase
51	um03973		related to serine/threonine protein kinase
52	um04136		related to serine/threonine protein kinase
53	um04258	ubc4	MAPKK kinase
54	um04456	adr1	protein kinase A, catalytic subunit
55	um04539		related to POS5 - Mitochondrial NADH kinase
56	um04543		related to Protein kinase lkh1
57	um04659		probable glycerol kinase
58	um04755		related to calcium/calmodulin dependent protein kinase C
59	um04808		related to serine/threonine protein kinase
60	um04871	pgk1	probable PGK1 - phosphoglycerate kinase
61	um04901		related to CKI1 - choline kinase
62	um04902	kin28	probable KIN28 - cyclin-dependent ser/thr protein kinase

Table S2. *Cont.*

No.	Entry	Gene	Description
63	um04925		related to CTK1 - carboxy-terminal domain (CTD) kinase, alpha subunit
64	um04931		related to PIK1 - phosphatidylinositol 4-kinase
65	um04950		related to cyclin dependent kinase C
66	um04956	ukc1/cbk1	protein kinase Ukc1p
67	um04962		related to ARG82 - dual-specificity inositol polyphosphate kinase required for regulation of phosphate- and nitrogen-responsive genes
68	um04991		related to Serine/threonine protein kinase
69	um05014		related to Serine/threonine kinase
70	um05022		related to PKH1 - ser/thr protein kinases
71	um05045		related to GCN2 - ser/thr protein kinase
72	um05130		probable phosphoenolpyruvate carboxykinase
73	um05216		related to IKS1 - putative serine/threonine kinase
74	um05275		related to pyruvate dehydrogenase kinase isoform 2, mitochondrial
75	um05543	don3	Ste20-like kinase Don3
76	um05544		probable calmodulin-dependent protein kinase type 1
77	um05698	nrc-2	probable ser/thr protein kinase
78	um05726		related to RIO Kinase 1
79	um05924	adk2	probable ADK2 - adenylate kinase, mitochondrial
80	um06019	cmk1	probable CMK1 - Ca2+/calmodulin-dependent ser/thr protein kinase type I
81	um06086		related to branched-chain alpha-ketoacid dehydrogenase kinase, mitochondrial precursor
82	um06103		related to phosphatidylinositol-4-kinase
83	um06107		related to casein kinase II beta subunit (regulator of circadian clock protein FRQ)
84	um06239		related to phosphatidylinositol 3-phosphate 5-kinase
85	um06306		related to YAK1 - ser/thr protein kinase
86	um06337	Wee1	wee1 kinase
87	um06383		related to MAP kinase
88	um06450	PkaR	cAMP-dependent protein kinase type II regulatory chain
89	um10056		related to serine/threonine-protein kinase
90	um10064		probable glucokinase
91	um10107		probable mitogen-activated protein kinase MpkA
92	um10119	ipl1	probable IPL1 - ser/thr protein kinase
93	um10123		probable Serine/threonine-protein kinase gad8
94	um10145	cla4	p21-activated kinase
95	um10206		related to Serine/threonine-protein kinase mph1
96	um10237		related to CDC7 - protein kinase
97	um10310		related to pyridoxal kinase
98	um10465.2		probable FAB1 - phosphatidylinositol 3-phosphate 5-kinase
99	um10496	kic1	related to KIC1 - ser/thr protein kinase that interacts with Cdc31p
100	um10598		probable nucleoside-diphosphate kinase
101	um10705	cdk1	cyclin-dependent kinase 1

Table S2. *Cont.*

No.	Entry	Gene	Description
102	um10720		related to ser/thr protein kinases
103	um10797		related to 6-phosphofructo-2-kinase
104	um10855		related to MKK1 - MAP kinase kinase
105	um10979		related to pantothenate kinase
106	um10985	thr1	probable THR1 - homoserine kinase
107	um10999		related to NRK1 - Nicotinamide riboside kinase
108	um11041.2		related to serine/threonine-protein kinase
109	um11087		related to checkpoint kinase chk1
110	um11125	ura6	probable URA6 - uridine-monophosphate kinase
111	um11141		related to FMN1 - Riboflavin kinase
112	um11195		related to serine/threonine-specific protein kinase KIN1
113	um11199	cdc28	related to CDC28 - cyclin-dependent protein kinase
114	um11293		related to SNF1 - carbon catabolite derepressing ser/thr protein kinase
115	um11296.2		related to calmodulin-dependent protein kinase
116	um11361.2		related to branched chain alpha-ketoacid dehydrogenase kinase
117	um11396	Nak1	related to ser/thr protein kinase
118	um11409		probable 6-phosphofructokinase
119	um11410	Crk1	cdk-related kinase 1
120	um11677.2		related to serine/threonine protein kinase
121	um11739		related to Nucleoside diphosphate kinase 6
122	um11808		related to LCB5 - sphingolipid long chain base kinase
123	um11860		cAMP-dependent protein kinase catalytic subunit
124	um11892	Pho85	probable PHO85 - cyclin-dependent protein kinase
125	um11912		related to mRNA splicing-associated serine-threonine protein kinase
126	um11945		probable hexokinase
127	um11957		related to histidine kinase
128	um12041		related to LSB6 - Phosphatidylinositol 4-kinase
129	um12089		related to p53-related protein kinase
130	um12177	Xks1	probable XKS1 - xylulokinase
131	um12208		related to SSN3 - cyclin-dependent CTD kinase
132	um12272	Smu1	Ste20-like protein kinase; has effect on mating
133	um15015		related to putative dual specificity protein kinase pom1 (C-terminal fragment)
134	um15023		probable protein kinase C
135	um15092		probable PBS2 - tyrosine protein kinase of the MAP kinase kinase family
136	um15093		related to Serine/threonine-protein kinase

In the genome of *U. maydis* 136 genes for kinases have been identified. Kinases used in the course „Chemical genetics“ are marked in yellow. The entry numbers are according to the *U. maydis* database MUMDB (http://mips.helmholtz-muenchen.de/genre/proj/ustilago/).
